# Carrier-mediated transport as a common route of antibiotic ingress into bacteria

**DOI:** 10.1128/mbio.01616-25

**Published:** 2025-07-09

**Authors:** Justin E. Clarke, Luiza H. Galarion, Abdulmenem A. Almadani, Christopher N. A. Smith, John D. Wright, Stuart L. Warriner, Alex J. O'Neill

**Affiliations:** 1 Astbury Centre for Structural Molecular Biology, University of Leeds 684905 , Leeds, United Kingdom; 2 School of Molecular & Cellular Biology, Faculty of Biological Sciences, University of Leeds 339728 , Leeds, United Kingdom; 3 School of Chemistry, University of Leeds 4468, Leeds, United Kingdom; McMaster University, Hamilton, Ontario, Canada

**Keywords:** antibiotic accumulation, drug uptake, transporters, import, importer

## Abstract

Among the most significant challenges in antibacterial discovery is achieving delivery of small molecule inhibitors across the bacterial membrane(s) to reach their intracellular targets. It is considered axiomatic that the typical route for antibiotics across the cytoplasmic membrane (CM) involves diffusion directly through the lipid bilayer, though this concept has not translated into actionable information for rationally achieving ingress of inhibitors into bacteria. Here, using antibiotic accumulation measurements in bacteria inactivated for individual or multiple CM transporters, we provide evidence that a diverse range of established antibiotic classes are transported across the CM by hitch-hiking on carrier proteins. The chemical similarity between some antibiotic classes and the native substrate(s) of the importer they share provides a molecular logic for drug transport. Our results indicate that carrier-mediated uptake is a common route of antibiotic entry into bacteria and highlight the value of metabolite mimicry in designing new antibacterial drugs.

**IMPORTANCE:**

Increasing antibiotic resistance among pathogenic bacteria is undermining our ability to treat infection, and new antibacterial drugs are urgently needed to address the problem. One of the most significant challenges in antibacterial discovery is achieving delivery of inhibitors across the bacterial membrane(s) to reach their intracellular targets, reflecting the lack of a granular understanding about how antibiotics enter bacteria. Here, we provide evidence that a common route for established antibiotics into bacteria involves uptake by membrane transport proteins. Our findings thereby offer a clear route forward for antibacterial discovery: mimicry of the natural substrates of transporter proteins to achieve carrier-mediated uptake of inhibitors into the bacterial cell.

## INTRODUCTION

Increasing antibiotic resistance in bacterial pathogens is eroding the therapeutic utility of our existing antimicrobial armamentarium, and new drugs are urgently required to address the problem. Unfortunately, that is proving easier said than done—the field of antibacterial drug discovery is now >35 years into a “discovery void,” a period during which no new classes active against the common bacterial pathogens have successfully progressed from discovery to clinic (1).

Among the most significant issues hindering antibacterial drug discovery is a lack of understanding about how to deliver small molecule inhibitors into bacteria (2–5). The majority of drug targets in bacteria are intracellular, and inhibitors aimed at such targets must therefore traverse the bacterial membrane(s) as a prelude to exerting their antibacterial effect. That getting inhibitors into bacteria constitutes a considerable challenge was not apparent throughout much of the history of antibacterial discovery; the traditional screening approach that employs growth inhibition of indicator bacteria as the readout (i.e., direct detection of an antibacterial effect) will by default only return hits that are able to reach their cellular targets. However, with the widespread adoption of target-based discovery—which typically involves screening for inhibitors against a purified molecular target of interest—this issue has come to the fore. It is by now relatively straightforward to identify or create potent inhibitors of purified bacterial enzymes using the tools of modern drug discovery (4, 6). Nevertheless, such inhibitors typically lack activity against intact bacteria because they cannot cross bacterial membranes, and attempts to imbue these compounds with improved cell-penetrating properties through chemical modification are usually unsuccessful. Indeed, this failure to convert target inhibitors to antibacterial drug candidates represents the most frequent point of attrition in industrial antibacterial discovery programmes (5, 6).

There has been valuable progress in recent years in understanding the rules of compound permeation across the outer membrane (OM) of gram-negative bacteria (2, 7, 8). Since the OM is a major contributor to the intrinsic resistance that gram-negatives exhibit against many agents active against gram-positive bacteria (2, 9, 10), this information can inform the conversion of gram-positive-only antibacterials into broad-spectrum agents. Nevertheless, the current lack of a comparable understanding of small molecule permeation across the cytoplasmic membrane (CM) that is universally present in all bacterial pathogens prevents rational design from scratch of inhibitors capable of reaching the bacterial cytoplasm.

It is considered axiomatic that the typical route for clinically deployed antibiotics across the CM involves diffusion directly through the lipid bilayer (“passive lipoidal diffusion” [PLD]) (11, 12). Some isolated exceptions to this concept are recognized, with D-cycloserine and fosfomycin (FOS) shown instead to undergo transport across the CM by “hitchhiking” on carrier proteins (13). The starting point of the present study to better understand how antibiotics cross the CM was that the prevailing view of antibiotic ingress by PLD is probably incorrect and that carrier-mediated transport is more likely to be the main route by which such compounds traverse this membrane. This hypothesis was based on the idea that the latter is the typical route of small molecules across biological membranes more generally, a view that has been most articulately promulgated by Kell and colleagues (14–18). Several ideas and observations lend support to this concept, including the fact that many antibiotics do not display a suitable cLogP value for diffusion through a hydrophobic lipid bilayer (19), that robust experimental demonstration of PLD is lacking (14, 15), and that many natural small molecules—and even ions—have been shown to be dependent on carriers to cross membranes (20).

Here, we provide evidence that a diverse range of established antibiotic classes actually traverse the CM by carrier-mediated transport. Our findings imply that the solution to the antibiotic “ingress problem” will be to incorporate chemical features into inhibitors that mimic natural metabolites, thereby allowing them to hitch-hike into bacteria on the cognate transport proteins.

## MATERIALS AND METHODS

### Bacteria and plasmids

Bacterial strains and plasmids used in this study are described in Table 1. Plasmid pRN112 was a gift from Reindert Nijland (Addgene plasmid #84464; http://n2t.net/addgene:84464; RRID:Addgene_84464). *Escherichia coli* IM08B (LMBP 9582) and plasmid pIMAY-Z (LMBP 10259) were gifts from Tim Foster and are available from the Belgium Coordinated Collections of Microorganisms (http://bccm.belspo.be/).

**TABLE 1:** Bacteria and plasmids used in this study

Strain/plasmid	Description	Source
*S. aureus* JE2	Derivative of methicillin-resistant *S. aureus* isolate USA300_FPR3757 and parent strain of the NTML	(21)
*S. aureus* JE2_mAmetrine_	Fluorescent strain of JE2 generated using plasmid pRN112 (*below*) and used as a parent strain in competition growth assays	This study
Nebraska Transposon Mutant Library (NTML)	Comprises 1920 defined Tn insertion mutants of JE2	(22)
*E. coli* BW25113 *E. coli* JW2482 *E. coli* JW2850	Parent strain of the Keio collection; derivative of *E. coli* MG1655 Keio collection strain deleted for *uraA* Keio collection strain deleted for *xanQ*	(23) (24) (24)
*E. coli* JW2408	Keio collection strain deleted for *ptsH*	(24)
*E. coli* JW2409	Keio collection strain deleted for *ptsI*	(24)
*E. coli* IM08B	Derivative of *E. coli* DC10B; cloning host for modifying recombinant DNA to allow introduction into *S. aureus*	(25)
pRN112	Plasmid for chromosomal integration of gene encoding the fluorescent protein, mAmetrine, in *S. aureus*	(26)
pIMAY-Z	Plasmid for allelic exchange/ markerless deletion in *S. aureus*	(25)

### Antibiotic susceptibility testing

Minimum inhibitory concentrations (MICs) were determined according to established methodology (27). Briefly, bacteria were grown in cation-adjusted Mueller-Hinton broth (MHB-II) (Sigma-Aldrich) at 37°C with vigorous aeration for ~16 h. Cultures were diluted in fresh MHB-II to a cell density of ~5.5 × 10^5^ CFU/mL. An aliquot (90 µL) of the resulting suspensions was transferred to 96-well microtiter plates containing antibiotic (10 µL per well) across a range of concentrations. Standard MIC determinations used the typical twofold dilution series of antibiotic; for subsequent, more granular evaluation of susceptibility (“extended gradient susceptibility testing”), the concentration increments corresponded to 0.2× MIC of the antibiotic against the parent strain. Plates were sealed with gas-permeable moisture-barrier seals (Azenta Life Sciences) and incubated at 37°C with vigorous aeration for ~16 h. MIC values were determined as the lowest concentration of antibiotic at which the turbidity of the culture was <10% of the control culture containing no antibiotic.

### Competition growth assay

Bacteria were propagated overnight as described above. Cultures were diluted in fresh MHB-II to generate pairwise mixtures containing ~2.75 × 10^3^ CFU/ml each of the parent and the competitor strain. An aliquot (90 µl) of this mixed inoculum was transferred to the wells of 96-well microtiter plates containing either sterile water (control) or antibiotic across a range of concentrations (10 µl per well). Experiments typically employed antibiotics at increments of 0.2× of the MIC (relative to the parent strain), or—for screening—used final antibiotic concentrations of 0.5× and 0.7× MIC. Plates were sealed and incubated as above. For competition experiments using JE2_mAmetrine_ as the parent strain, cultures were grown in black, clear-bottom microtiter plates (Thermo Fisher) to enable fluorescence (FL) measurements (excitation 420 nm, emission 500 nm). FL values were normalized against OD_600_ to give FL/OD, and competition cultures showing a significant reduction (≥2-fold) in the FL/OD value in the presence of an antibiotic were taken to show out-competition of the parent strain by the mutant. For assays using the non-fluorescent JE2 strain, co-cultures were plated on selective and non-selective agar to enumerate mutant and total cells, respectively, following overnight growth at 37°C (in the case of *Staphylococcus aureus*, selective plates were tryptic soya agar with 5 µg/mL erythromycin, while for *E. coli*, Luria agar containing 25 µg/mL kanamycin was used).

### Antibiotic accumulation assays

Bacteria were cultured in MHB-II at 37°C with vigorous aeration for ~16 h, and ~2 × 10^9^ CFUs harvested by centrifugation at 4000 × *g* for 5 min. Supernatant was discarded, and bacteria were subjected to a second brief centrifugation to allow removal of residual media. Cells were resuspended in 1 mL of 0.1 M sodium phosphate buffer (pH 7) and incubated at 37°C with shaking for 10 min. Antibiotics were then added at the final concentrations detailed in the Results. Suspensions were incubated for a further 20 min before immersing in an ice bath for 1 min. Bacteria were harvested by centrifugation at 8,000 × *g*, 4°C for 2 min and the supernatant removed by aspiration. The surface of the resulting cell pellet was gently washed by submersion in ice-cold 0.1 M sodium phosphate buffer (pH 7), followed by immediate removal of the buffer.

For FL-based determination of ciprofloxacin accumulation, the method was adapted from previous work (28). Briefly, cell pellets were resuspended in 1 mL of 0.1 M glycine-HCl (pH 3) and incubated at room temperature overnight. Lysate was cleared by centrifugation at 16,000 × *g* for 10 min, and 900 µL of the supernatant transferred to a UV cuvette (Thermo Fisher) containing 1.1 mL ultrapure water. Samples were mixed and ciprofloxacin quantitated by scanning on an LS45 luminescence spectrometer (Perkin Elmer) with excitation at 280 nm and emission measured between 390 and 490 nm at 10 nm increments.

For accumulation measurements using liquid chromatography-mass spectrometry (LC-MS), cell pellets were resuspended in 1 mL ultrapure water and transferred to 2 mL tubes containing lysing matrix B (MP Biomedicals). Bacteria were lysed by homogenization on a FastPrep-24 homogenizer (MP Biomedicals) with two cycles of 6.5 M/S for 60 s, followed in each case by cooling on ice for 5 min. Lysate was cleared by centrifugation at 16,000 × *g* for 10 min, and 200 µL was mixed with 800 µL HPLC-grade acetonitrile (Thermo Fisher). Samples were vortexed and incubated on ice for 20 min, cleared by centrifugation (16,000 × *g* for 10 min), and 250 µL of the resulting supernatant mixed with 750 µL ultrapure water. Samples were filtered through 0.2 µm nylon syringe filters before subjecting them to LC-MS using an Acquity UPLC CSH C18 130 Å, 1.7 µm, 2.1 × 100 mm column (Waters) attached to a Dionex UltiMate 3000 UHPLC System (Thermo Fisher) with a mobile phase consisting of a gradient of up to 80% (vol/vol) acetonitrile, 0.1% (vol/vol) formic acid. Sample volume was set to 5 µL and run time to 3 min. Mass spectra were obtained on a maXis Impact oQTOF mass spectrometer (Bruker) via electrospray ionization. Extracted ion chromatograms (EIC) were generated based on the signal intensity of the appropriate *m*/*z* peaks. Integration of the EIC peaks was performed to obtain final peak areas, which were used to represent cytoplasmic concentration of antibiotic.

### Allelic exchange to delete genes encoding transport proteins

Markerless gene deletion utilized DNA fragments of ~2 kb in length, comprising fused ~1 kb regions corresponding to the up- and downstream sequence of genes targeted for deletion; these DNA fragments were obtained by synthesis (Genscript or Twist Bioscience) and ligated into plasmid pIMAY-Z (25). The resulting constructs were passaged in *E. coli* IM08B (29) before recovery and electroporation (30) into JE2. Transformants were recovered on TSA with 20 µg/mL chloramphenicol at 28°C. Allelic exchange was then performed as previously described (25). Gene deletions were confirmed via PCR using oligonucleotide primers flanking the sites of deletion.

## RESULTS

### Identification of the NCS-2 family permeases as ciprofloxacin importers in *S. aureus*

If an antibiotic ordinarily enters bacteria via a transport protein, inactivation of that transporter should cause a reduction in the rate and/or extent of intracellular antibiotic accumulation; where the resulting drop in accumulation is substantial, this effect should in turn manifest as a detectable reduction in antibiotic susceptibility. The starting point for this study was therefore to screen a transposon (Tn) insertion library in the model gram-positive bacterium *S. aureus* to detect transposants capable of growing at higher antibiotic concentrations than the parent strain, thereby allowing us to identify transport proteins involved in antibiotic ingress. Screening of Tn libraries for altered antibiotic susceptibility typically returns a great many “hits” (31–34); to reduce confounding and simplify the screening approach, we first established a bespoke library exclusively comprising members in which transporter genes are inactivated. Using Transport DB_2.0 (35), we identified 350 transporters in *S. aureus* USA300_FPR3757 at the time of analysis; of these, 318 have corresponding gene inactivation strains in the Nebraska Transposon Mutant Library (NTML) (22). These latter strains were recovered from the NTML to establish what is referred to hereafter as the SATKO library. The SATKO library was screened by spotting onto agar containing antibiotics at concentrations higher than those that completely inhibit growth of the parent strain (*S. aureus* JE2). Transposants that grew (“hits”) were then tested by broth microdilution to confirm reduced antibiotic susceptibility. This screening approach was validated using FOS, an antibiotic that is known to traverse the CM via two carrier proteins (the glycerol-3-phosphate transporter, GlpT, and the glucose-6-phosphate [G6P] transporter, UhpT) (13). The latter is only expressed in the presence of G6P (36, 37), and we did not therefore expect to detect the *uhpT* transposant under the growth conditions of our screen. As anticipated, only a single strain (NE1388) from the SATKO library grew on agar containing 8 µg FOS/mL, and FOS susceptibility testing gave an MIC of 128 µg/mL (16-fold higher than JE2); this transposant contains a disruption in *glpT* (SAUSA300_0337).

**Fig 1:**
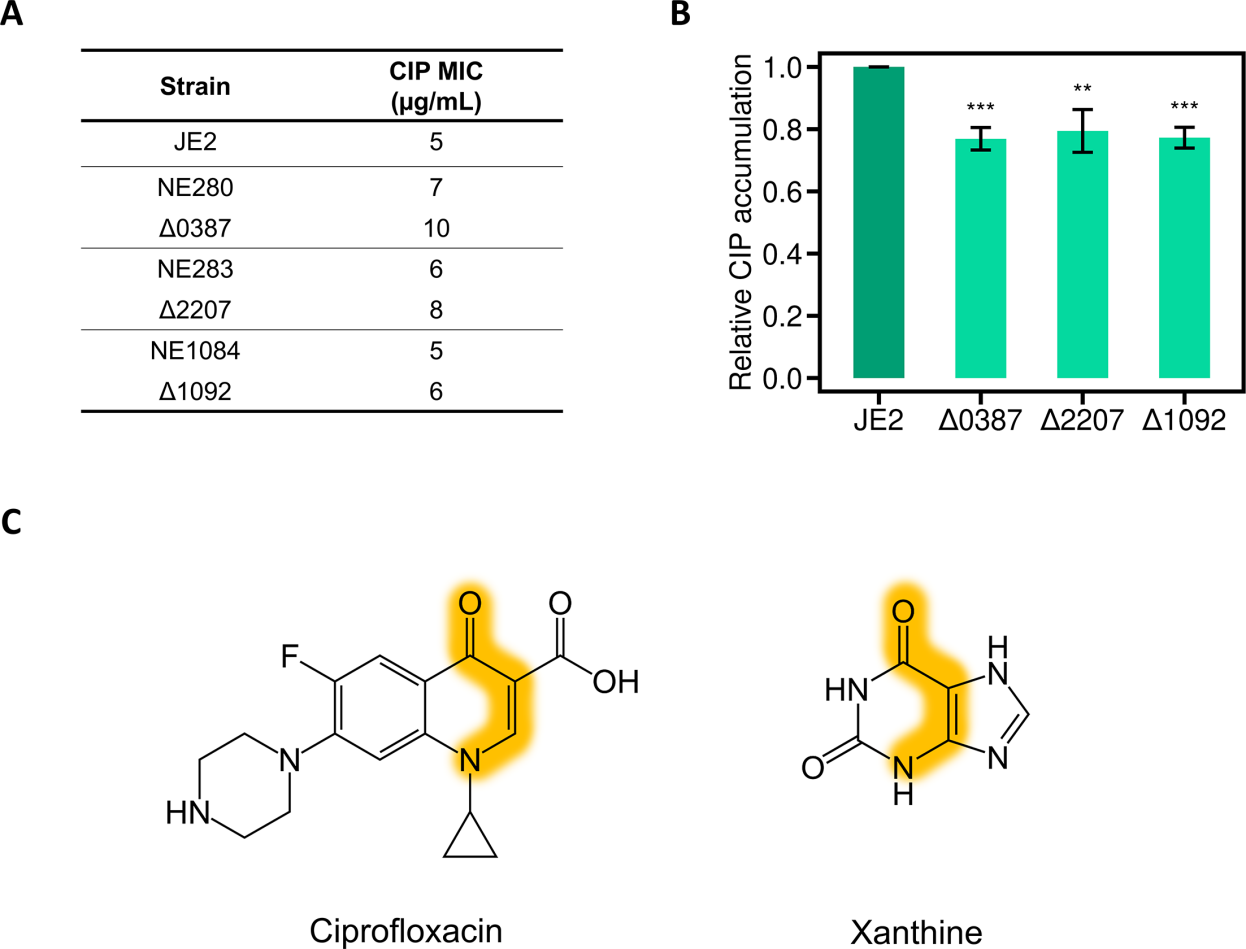
The NCS-2 family permeases mediate CIP uptake in *S. aureus*. (**A**) Independent inactivation of the three NCS-2 permeases in *S. aureus* JE2 confers reduced CIP susceptibility in each case. Results are shown for transposon insertion mutants (prefix “NE”) and the corresponding markerless deletion strain immediately below. Determinations were performed in triplicate. (**B**) Intracellular accumulation of CIP in JE2 independently deleted for NCS-2 genes, as determined using FL measurements. CIP was added at a final concentration of 4 µg/mL, and values are expressed relative to JE2 with error bars showing standard deviations across three independent experiments. Statistical significance in relation to the JE2 control was determined using an unpaired *t*-test, with ** denoting *P* < 0.01 and *** denoting *P* < 0.001. (**C**) Maximum common substructure (MCS; highlighted in yellow) between CIP and a natural NCS-2 permease substrate, xanthine, with a Tanimoto score (MCS_min_) of 0.55 (calculated using ChemMine Web tools [38]).

We initially screened the SATKO library on agar containing ciprofloxacin (CIP) at a concentration of 8 µg/mL (above the MIC of 5 µg/mL). The two hits that resulted (NE280 and NE283) contain disruptions in the genes SAUSA300_0387 and SAUSA300_2207, respectively, both of which encode permeases of the NCS-2 sodium/proton:nucleobase symporter family. Extended-gradient susceptibility testing detected modest but reproducible increases in CIP MIC (i.e., reduced CIP susceptibility) in both cases (Fig. 1A). To corroborate this result and exclude the possibility that it was attributable to polar effects related to Tn insertion, we generated independent markerless deletions of SAUSA300_0387 and SAUSA300_2207 in JE2. In addition, we also generated a strain deleted for SAUSA300_1092, a third member of the NCS-2 family present in *S. aureus*; although the transposant (NE1048) carrying a disruption in this latter gene did not show reduced CIP susceptibility, we suspected that polar effects from Tn insertion might be obscuring the phenotype (the Tn in this strain resides in the first gene of an octacistronic operon, and the transposant appeared very unfit). All three NCS-2 transporter deletion strains showed a reproducible increase in CIP MIC, and in each case, the increase was greater than that seen for the corresponding transposant (Fig. 1A). To further confirm that the NCS-2 family permeases are involved in uptake of CIP into *S. aureus*, we exploited the intrinsic FL of CIP to measure intracellular accumulation of the drug in the deletion strains relative to the JE2 parent strain. All three NCS-2 deletion strains showed a reproducible reduction of between 10% and 30% in intracellular CIP accumulation compared to JE2 (Fig. 1B). Chemical similarity comparisons indicate that CIP has features in common with natural substrates of these importers (xanthine/uracil) (Fig. 1C), suggesting that CIP is recognized by these transporters owing to similar chemical features/through similar contacts to that seen for the natural substrates.

### A competition growth assay identifies importers for diverse antibiotic classes

**Fig 2:**
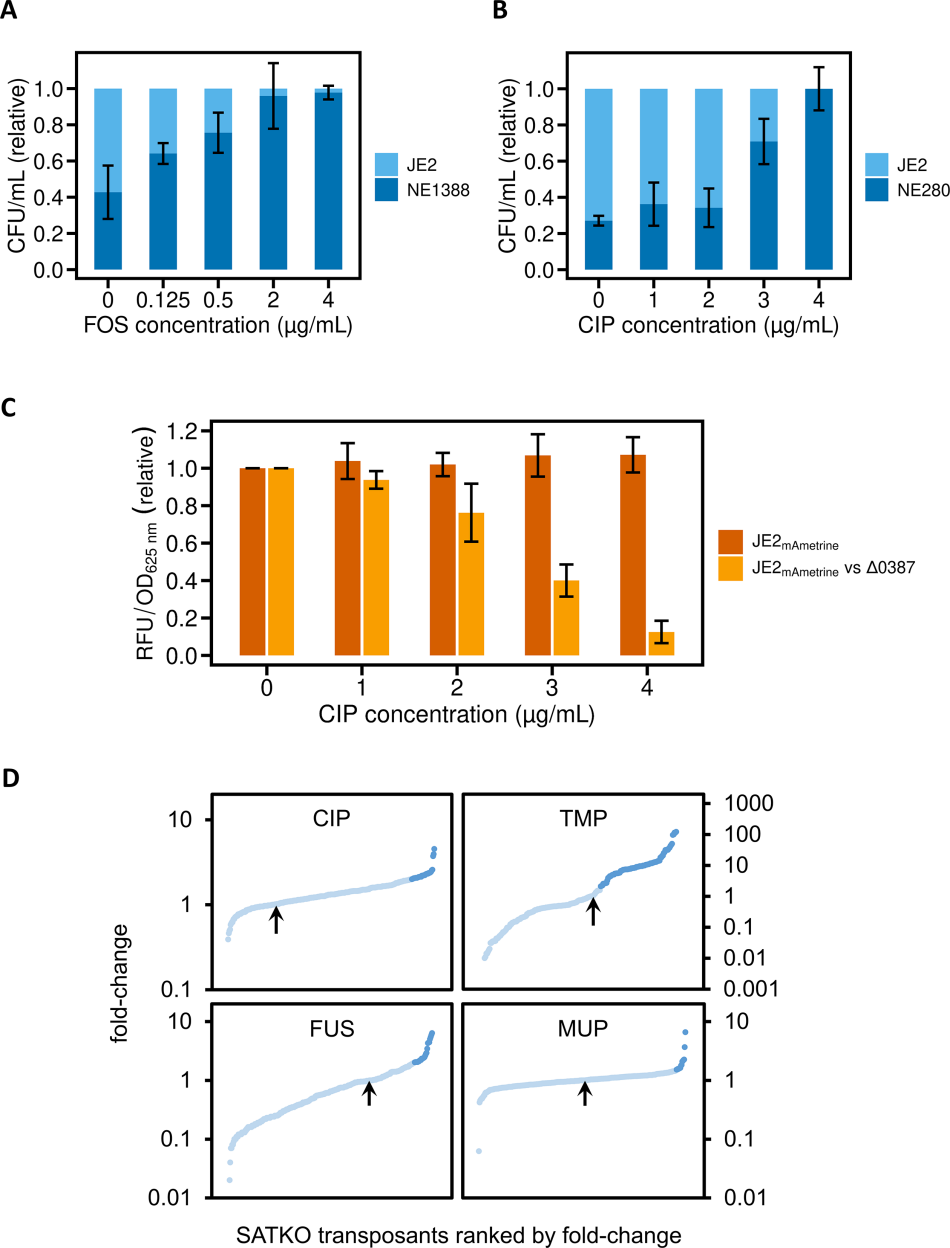
Competition growth assays to identify antibiotic importers in *S. aureus*. Panels A–C summarize key validation experiments for this approach; in all cases, error bars display standard deviations for three independent experiments. (**A**) Competition growth assays between *S. aureus* JE2 and a strain (NE1388) inactivated for the known FOS importer, *glpT*, show that the latter has a competitive advantage in the presence of FOS. (**B**) Likewise, a strain (NE280) inactivated for a CIP importer (SAUSA300_0387) identified in this study shows a competitive advantage over JE2 in the presence of CIP. (**C**) Competition growth experiments using the fluorescent strain JE2_mAmetrine_ in place of JE2 allow the competitive advantage of strains inactivated for antibiotic importers to be detected in a FL-based assay; the competitive advantage of a strain deleted for a CIP importer (ΔSAUSA300_0387) over JE2_mAmetrine_ in the presence of CIP results in a marked reduction in FL signal. (**D**) Competition growth screens of the SATKO library in the presence of different antibiotics. Each graph shows in rank order the fold change in relative cell number of each transposant compared to the parent (JE2_mAmetrine_) in each co-culture upon incubation with sub-MIC concentrations of CIP, trimethoprim (TMP), fusidic acid (FUS), or mupirocin (MUP). Dark-blue dots represent strains that showed a ≥2-fold increase in competitive advantage over the parent in the presence of antibiotics; these “hits” were considered to be strains in which an antibiotic importer has been inactivated and were therefore taken forward for further characterization. The arrows indicate in each case the negative control, which contained the non-fluorescent JE2 strain as the competitor.

Having gained evidence to support the idea that CIP enters *S. aureus* by carrier-mediated transport, we sought to assess whether this is also true of other antibiotics that are generally held to cross the CM by PLD. However, failure to recover reproducible hits for several antibiotics using the above approach (data not shown) prompted us to seek a more sensitive screening method. Competition growth assays have previously been used to identify transporters involved in the uptake of cytotoxic drugs in eukaryotic cells (18, 39), by virtue of the fact that strains lacking a relevant importer can outcompete the parent in mixed culture in the presence of the drug; the added element of competition in such an assay brings increased resolution over simply testing for the ability to grow in the presence of the drug. We therefore adopted a similar approach. In pilot experiments, we established that NE1388 (transposant inactivated for the FOS importer, *glpT*) exhibits a clear fitness advantage over the JE2 parent strain in the presence of FOS at concentrations as low as 1/64 of the FOS MIC against JE2 (Fig. 2A). We also confirmed that NE280 (transposant inactivated for the NCS-2 transporter, SAUSA300_0387) outcompeted the parent strain in the presence of CIP (Fig. 2B); however, in contrast to the situation seen with FOS, the fitness advantage in the latter case was observed only at CIP concentrations close to the MIC. To circumvent the labor-intensive plating/visual enumeration of bacteria to measure competition in the assay, a FL-based approach was established. The JE2 strain was engineered to carry a chromosomal copy of the gene encoding fluorescent protein mAmetrine using the vector pRN112 (26), thereby allowing competition to be monitored in mixed culture by measuring FL (a readout of parent strain population density) in relation to optical density (a readout of total culture density, corresponding to parent + competitor). Cultures of JE2_mAmetrine_ alone showed reproducible FL that did not decrease significantly in the presence of increasing concentrations of CIP, suggesting that the FL signal provides a robust measure of the number of parent cells in the culture (data not shown). Co-cultures of JE2_mAmetrine_ and the CIP importer deletion strain ΔSAUSA300_0387 showed a noticeable reduction in FL at 2–3 µg/mL CIP (Fig. 2C). This is a similar trend to that found when directly enumerating the number of parental and mutant cells on agar (Fig. 2B), implying that the FL assay is a suitable method to measure competition.

The competition growth assay was then used to screen the entire SATKO library with a cross-section of structurally distinct antibacterial drug classes that have intracellular targets (fusidic acid [FUS], mupirocin [MUP], and trimethoprim [TMP]); additionally, screening with CIP was performed to identify any importers missed in the original agar-based screen. The fold change in the number of mutant cells compared to parent cells when growing in the presence of an antibiotic was calculated for each co-culture and represented as ranked plots (Fig. 2D). Putative hits were identified as mutants exhibiting ≥2-fold increase in cell density compared to the parent in the presence of antibiotic and subsequently underwent further evaluation in the form of antibiotic susceptibility tests and LC-MS-based antibiotic accumulation assays.

Candidate transporters were identified for all four drugs tested (Table 2). While pronounced reductions in susceptibility were seen for some CIP and TMP hits (up to twofold and 6.7-fold increase in MIC, respectively) (Table 2), most of the hit strains did not show a detectable change in MIC using extended-gradient susceptibility testing. This latter observation is consistent with the results of the initial agar screen, from which only two transposants showed an increase in CIP MIC, and is considered further below. Nevertheless, the majority of hit strains exhibited a reduction in intracellular drug accumulation, seen for all of the FUS and MUP hits (9 and 6, respectively), 9/17 TMP hits and 3/6 of CIP hits (Table 2).

**TABLE 2:** Antibiotic susceptibility and accumulation for hit transposants from the SATKO library that exhibited increased competitive fitness in the presence of antibiotics^*a*^^,^^*b*^

Antibiotic	Strain	Disrupted gene	Natural substrate(s) of encoded transporter	MIC (µg/mL)	Antibiotic accumulation (relative to parent)
CIP	JE2	–	–	5	1
	NE333	SAUSA300_0209	Maltose	6	1.13
	NE663	SAUSA300_0211	Maltose	5	0.94
	NE771	SAUSA300_2379	Unknown	10	1.13
	NE810	SAUSA300_1642	D-serine/alanine	5	0.97
	NE1030	SAUSA300_2628	Unknown	5	1
	NE1380	SAUSA300_0978	Thiamine	6	0.85
TMP	JE2	–	–	1.5	1
	NE187	SAUSA300_0650	Phosphate	2.5	1.02
	NE566	SAUSA300_2329	Glutamate	1.5	0.94
	NE568	SAUSA300_2351	Zinc	1.5	0.96
	NE658	SAUSA300_1002	Spermidine/putrescine	2	1
	NE734	SAUSA300_1978	Iron-hydroxamate	2	1.17
	NE1083	SAUSA300_1001	Spermidine/putrescine	1.5	0.82
	NE1115	SAUSA300_2434	Unknown	2.5	0.99
	NE1159	SAUSA300_0999	Spermidine/putrescine	2.5	0.86
	NE1229	SAUSA300_1515	Manganese	1.5	1.01
	NE1376	SAUSA300_2323	Cobalt	1.5	1.07
	NE1541	SAUSA300_2233	Biotin	1.5	1.17
	NE1580	SAUSA300_1191	Glycerol	2	0.93
	NE1592	SAUSA300_2359	Glutamine/ectoine	10	1
	NE1629	SAUSA300_2618	Riboflavin/thiamine	2	0.92
	NE1751	SAUSA300_0979	Thiamine	10	1.16
	NE1765	SAUSA300_1187	Thiamine	1.5	0.95
	NE1823	SAUSA300_0345	Iron	2.5	0.94
FUS	JE2	–	–	0.2	1
	NE34	SAUSA300_2313	Lactate	0.2	0.96
	NE39	SAUSA300_2476	GlcNAc	0.2	0.71
	NE344	SAUSA300_2103	Molybdate	0.2	0.86
	NE392	SAUSA300_0200	D-methionine	0.2	0.94
	NE1068	SAUSA300_2552	Citrate	0.2	0.83
	NE1125	SAUSA300_0064	Arginine/ornithine	0.2	0.79
	NE1131	SAUSA300_2395	Amino acid	0.2	0.84
	NE1314	SAUSA300_0896	Oligopeptide	0.2	0.74
	NE1706	SAUSA300_1870	Fusaric acid (efflux)	0.2	0.81
MUP	JE2	–	–	0.15	1
	NE142	SAUSA300_1252	Alanine	0.15	0.82
	NE280	SAUSA300_0387	Xanthine	0.15	0.90
	NE810	SAUSA300_1642	D-serine/alanine	0.15	0.84
	NE891	SAUSA300_0924	Potassium	0.15	0.98
	NE945	SAUSA300_0188	Branched amino acid	0.15	0.87
	NE1751	SAUSA300_0979	Thiamine	0.15	0.84

^*a*^ Antibiotic accumulation of CIP (4 µg/mL), TMP (3 µg/mL), MUP (3 µg/mL) and FUS (1 µg/mL) was quantified by LC-MS and is presented as the means of ≥2 determinations.

^*b*^ "–" indicates that there is nothing to report.

### Inactivating multiple antibiotic importers in a single strain further reduces antibiotic ingress

**Fig 3:**
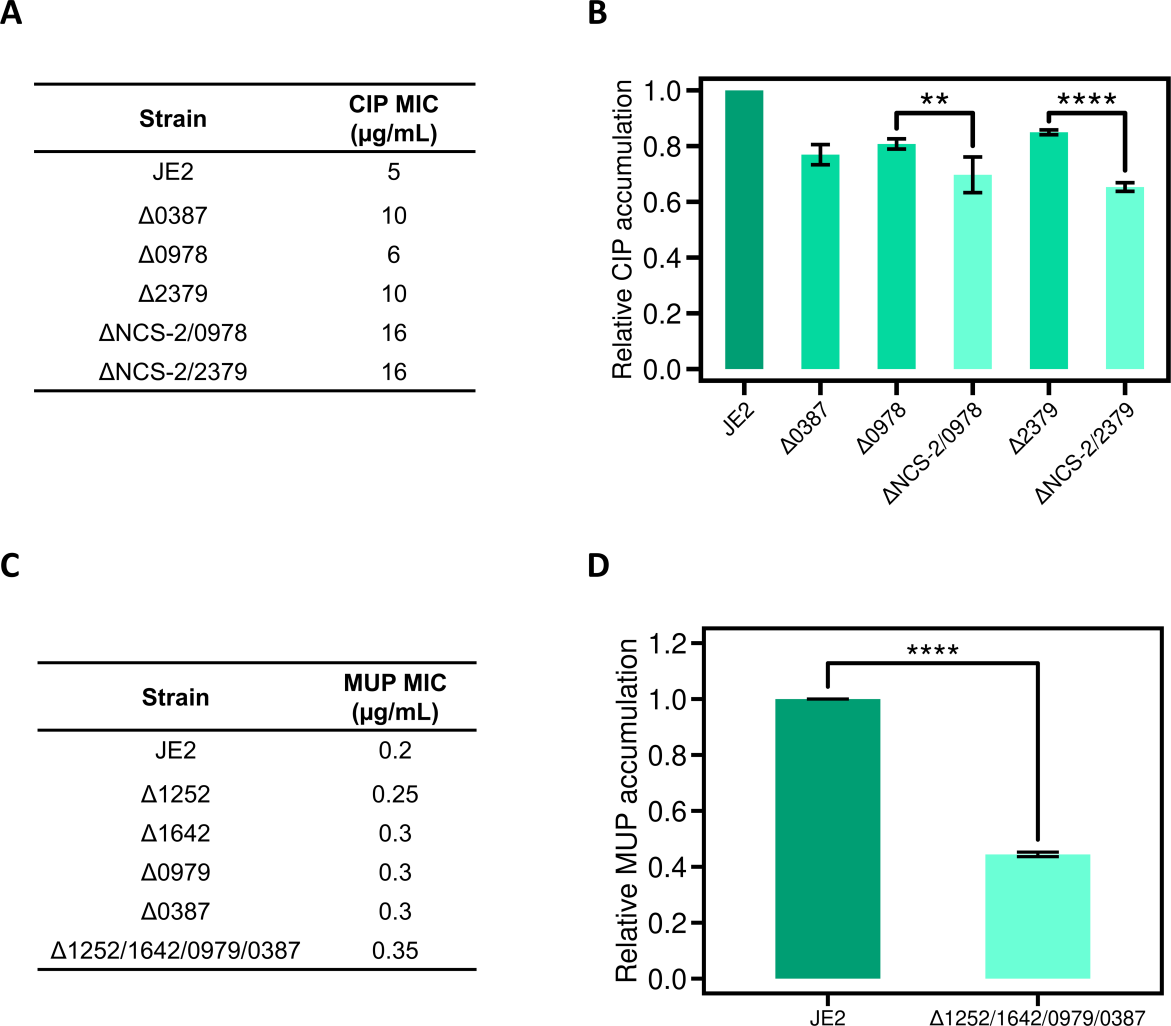
Deleting multiple antibiotic importers in a single strain further reduces antibiotic ingress into the cell. (**A**) CIP susceptibility of JE2 strains deleted for single and multiple CIP importers. “ΔNCS-2” denotes deletion of all three members of the NCS-2 family (SAUSA300_0387, SAUSA300_1092, and SAUSA300_2207). Determinations were performed a minimum of three times to ensure reproducibility. (**B**) Intracellular accumulation of CIP in strains deleted for CIP importers. CIP was added at a final concentration of 4 µg/mL, and accumulation was quantified using fluorescent measurements. For comparison, data for the ΔSAUSA300_0387 strain are also shown from Fig. 1B. Error bars display standard deviations for three independent experiments. Significance values were determined using an unpaired *t*-test, with ** denoting *P* < 0.01 and **** denoting *P* < 0.0001. (**C**) MUP susceptibility of JE2 strains deleted for single and multiple MUP importers. Determinations were performed a minimum of three times to ensure reproducibility. (**D**) Intracellular accumulation of MUP in strains deleted for MUP importers. MUP was added at a final concentration of 20 µg/mL, and accumulation was quantified using LC-MS. Error bars display standard deviations for three independent experiments, with significance reported as for panel **B**.

The identification of multiple importers for each antibiotic screened implies redundancy in the routes by which these compounds enter *S. aureus*. This accords with observations made regarding small molecule transport in eukaryotes (40, 41) and provides an explanation for why the phenotypic impact (reduction in antibiotic susceptibility/ accumulation) observed here for strains lacking a single antibiotic transporter is typically modest (Table 2). To corroborate and further explore this idea, we generated individual strains in which multiple transporters for a given antibiotic were deleted. Initially, we focused on CIP importers, constructing two quadruple deletion strains that lacked all three of the NCS-2 permease genes (SAUSA300_0387, 2207, and 1092) and either the SAUSA300_0978 or the SAUSA300_2379 gene. Both quadruple deletion strains were associated with a CIP MIC of 16 µg/mL, a substantial increase over strains carrying single deletions of these same genes (6–10 µg/mL) and a >3-fold increase compared to the MIC of 5 µg/mL for the JE2 parent strain (Fig. 3A). The quadruple deletion strains also showed a more pronounced effect on CIP accumulation; for both of these strains, accumulation dropped to ~65% relative to JE2, in contrast to the ~80% CIP accumulation for single deletion strains (Fig. 3B).

A comparable experiment was performed for MUP importers. Strains harboring single clean deletions of SAUSA300_1252, SAUSA300_1642, SAUSA300_0979, and SAUSA300_0387 were evaluated alongside a strain deleted for all four of these genes for their MUP susceptibility and accumulation. The effect on MUP susceptibility was modest, with each of the single deletions associated with an increase in MIC from 0.2 to 0.3 µg/mL; however, the quadruple deletion strain reproducibly showed a further increase in MUP MIC to 0.35 µg/mL (Fig. 3C). More strikingly, the quadruple deletion strain showed greatly reduced accumulation of MUP down to 45% that of the parent strain (Fig. 3D), considerably lower than seen for strains in which these transporters were individually inactivated (82%–90%; Table 2)

### Evidence for carrier-mediated uptake of antibiotics into *E. coli*

To support the generalizability of the concept of carrier-mediated antibiotic uptake to other bacteria, we examined this phenomenon in *E. coli*, a model gram-negative bacterium separated from *S. aureus* by >2 billion years of evolution (42). While *E. coli* mutants lacking transporters have been reported to show improved growth relative to the parent strain in the presence of antibiotics (32), the study in question did not establish that this effect was the result of reduced antibiotic accumulation.

**Fig 4:**
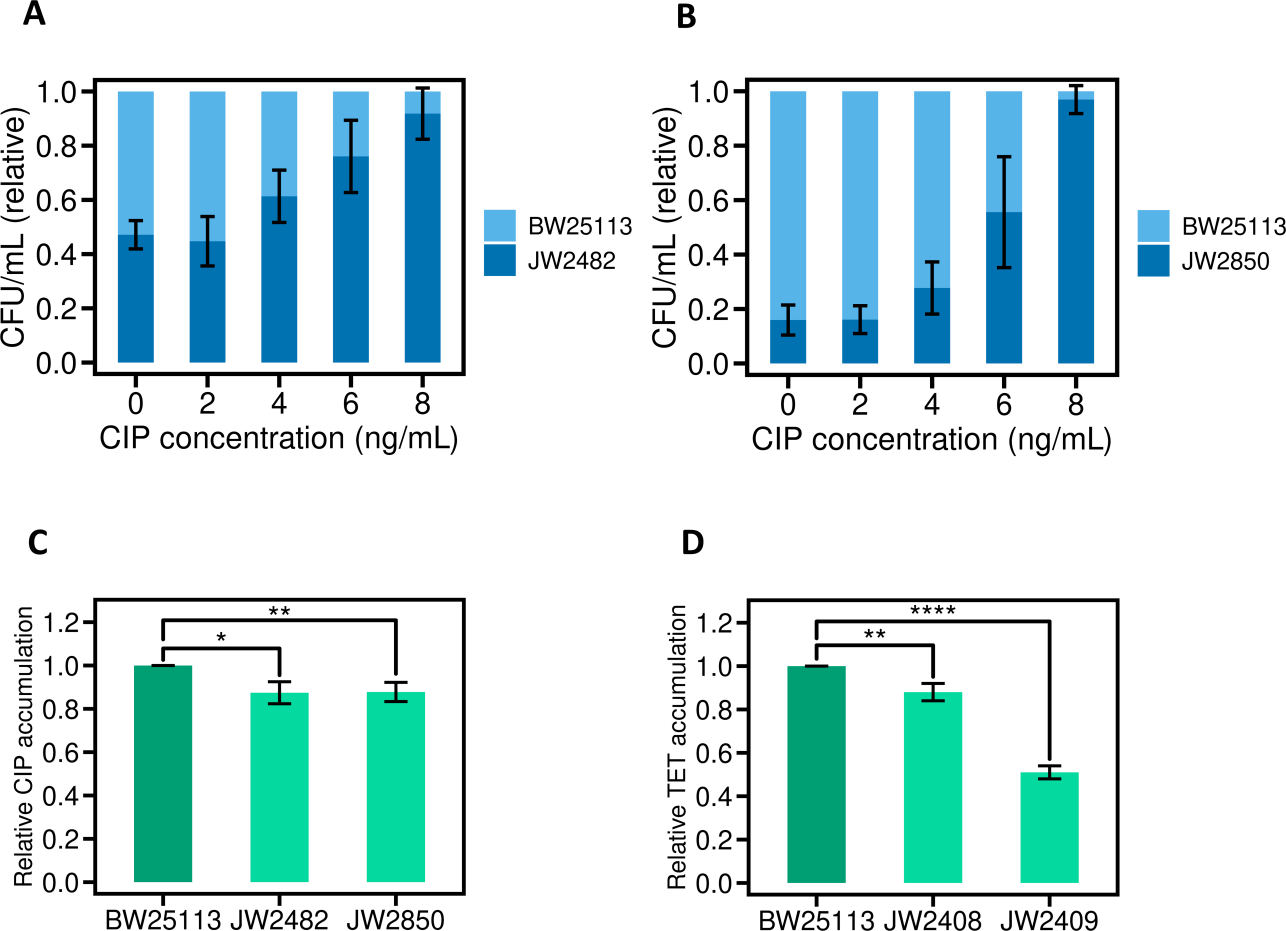
CM transporters mediate antibiotic import in *E. coli*. (**A**) Deletion of the NCS-2 permease gene, *uraA*, from *E. coli* BW25113 yields a strain (JW2482) that outcompetes BW25113 in the presence of CIP. (**B**) Likewise, a derivative (JW2850) of *E. coli* BW25113 deleted for another NCS-2 permease gene, *xanQ*, also outcompetes BW25113 in the presence of CIP. (**C**) Accumulation studies in JW2482 and JW2850 show reduced CIP accumulation relative to BW25113. CIP was added at a final concentration of 30 µg/mL and intracellular CIP accumulation quantified using LC-MS. (**D**) Reduced relative TET accumulation in two derivatives (JW2408 and JW2409) of *E. coli* BW25113 that carry deletions in a putative TET import system (PTS). TET was added at a final concentration of 20 µg/mL, and accumulation was quantified using LC-MS. For all experiments, error bars represent standard deviations for three independent experiments; for accumulation experiments, significance values were determined using an unpaired *t*-test (* is *P* < 0.05; ** is *P* < 0.01; **** is *P* < 0.0001).

In the first instance, we examined whether—as for *S. aureus*—the NCS-2 permeases in *E. coli* have a role in CIP uptake. The nucleobase permeases encoded by *uraA* and *xanQ* were chosen for this analysis as they represent the closest orthologs of the NCS-2 family in *S. aureus*, and both are well studied (43–46). Competition growth assays performed with *E. coli* BW25113 and direct derivatives independently deleted for these permease genes showed a competitive advantage for both of the latter in the presence of CIP (Fig. 4A and B), implying that CIP also traverses the CM via NCS-2 permeases in this organism. To confirm this, CIP accumulation assays were undertaken, which revealed a significant decrease in the accumulation of CIP relative to the parent strain in both cases (Fig. 4C).

We subsequently established an *E. coli* competition growth assay for transporter deletion mutants of the Keio collection (24), the nature and deployment of which will be described in a future publication. However, a preliminary screen for mutants showing a competitive advantage over the parent strain in the presence of the antibiotic tetracycline (TET) yielded two hits (Keio collection strains JW2408 and JW2409) that are deleted for genes encoding components of the phosphotransferase system involved in carbohydrate uptake: *ptsH* and *ptsI*, respectively. Subsequent accumulation experiments confirmed that loss of the phosphotransferase system is associated with reduced intracellular TET accumulation (Fig. 4D).

## DISCUSSION

Carrier-mediated transport of antibacterial drugs into bacteria is known to occur (13) but has to date been considered a rare exception to the “rule” of antibiotic entry via PLD. Here, we have provided evidence that five distinct classes of antibacterial drug cross the CM by hitch-hiking on transporter proteins, revealing carrier-mediated transport as a common route of such compounds into bacteria. Indeed, the identification of putative transporters for all of the drug classes examined here suggests that carrier-mediated transport is the predominant route by which antibiotics cross the CM.

For the antibiotics studied, we typically identified multiple transporters that mediate their import into bacteria. This redundancy accounts for the fact that inactivation of a given antibiotic importer generally has only a (very) modest impact on antibiotic susceptibility/accumulation; loss of only a single route into the cell would not be expected to yield a substantial reduction in antibiotic ingress when multiple other entry points remain. Furthermore, this redundancy in importers for a given antibiotic explains why bacteria have not typically been able to evolve clinical resistance to these agents through inactivation of transporter proteins and why the phenomenon of carrier-mediated transport has remained obscured for many antibiotic classes. As described above, FOS and D-cycloserine have long been considered unusual among clinically deployed antibiotics in that they enter bacteria via carrier proteins (13). Our results imply that these agents are not exceptions in their mode of entry into bacteria *per se* but by virtue of the fact that they hitch-hike on only a limited number of carriers; this lack of redundancy means that inactivation of a single importer is associated with a profound reduction in antibiotic susceptibility, which has in turn meant that this phenomenon was readily detected for these drugs and has provided a route by which resistance can evolve in clinical strains.

Some of the antibiotic importers identified in this study support the intuitive notion that drugs that hitch-hike into the cell on carrier proteins do so through mimicry of the native substrates (47–49), thereby permitting uptake via the same mechanism. As shown, CIP is imported via the NCS-2 family of symporters and displays chemical similarity with the natural substrate of these transporters (Fig. 1). Complementing this observation, we found that the concentrative nucleoside transporters (CNTs)—which transport similar substrates to the NCS-2 family—are not involved in the uptake of CIP (data not shown). This can be explained by the fact that CNT transporters recognize their nucleoside substrates through primary contacts with the ribose ring rather than the nucleobase group (50, 51); given that CIP only has chemical similarity to the latter, it would be unable to bind to CNTs. Another example of metabolite mimicry as the basis for hitchhiking on carrier proteins is seen for TMP, with the drug sharing both importers and a clear structural similarity with thiamine (Table 2; Fig. S1).

In many other cases, we did not detect obvious chemical similarity between an antibiotic and the native substrate(s) of an importer. This could reflect the fact that there are other considerations beyond chemical similarity at play, but we suspect that it is often simply that current knowledge of the range of substrates a given transporter can recognize is insufficient to reveal underlying chemical similarity to transporter substrates. For example, the xanthine/uracil permease encoded by SAUSA300_0387 that acts as a CIP importer also transports MUP (Table 2). While there is no obvious chemical similarity between MUP and xanthine/uracil, crystallographic data of the *E. coli* ortholog UraA has revealed that a cavity adjacent to the pore region can accommodate a nonyl β-D-glucopyranoside (45), a compound that was present as a detergent during crystallographic trials and that bears strong similarity to MUP.

In summary, our work shows that antibiotics commonly hitch-hike on transporters to cross the CM into bacteria. Our findings have considerable implications for antibacterial drug discovery and design and indicate that leveraging mimicry of metabolites for which CM importers exist will be key to developing new antibiotics capable of accumulating inside bacteria.

## SUPPLEMENTAL MATERIAL

Figure S1 mbio.01616-25-s0001.pdf Trimethoprim recognition by the thiamine uptake system.

ASM does not own the copyrights to Supplemental Material that may be linked to, or accessed through, an article. The authors have granted ASM a non-exclusive, world-wide license to publish the Supplemental Material files. Please contact the corresponding author directly for reuse.
