# Supplementary material for: Carrier-mediated transport as a common route of antibiotic ingress into bacteria
Source: mBio. 2025 Jul 9;16(8):e01616-25. doi: 10.1128/mbio.01616-25 (PMC12345173; doi:10.1128/mbio.01616-25)
Supplement: Figure S1 — Trimethoprim recognition by the thiamine uptake system. [file mbio.01616-25-s0001.pdf]

A

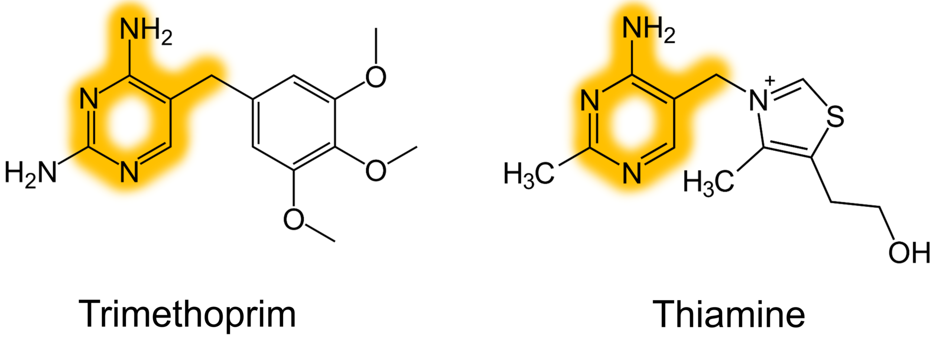

B

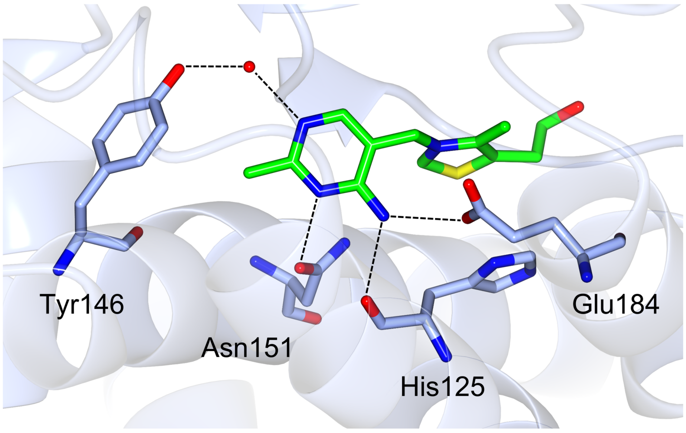

Figure S1. Trimethoprim recognition by the thiamine uptake system. (A) The maximum common substructure (MCS) between trimethoprim and thiamine is highlighted in yellow, with a Tanimoto score (MCS_{min}) of 0.44. (B) The crystal structure of thiamine (green) bound to ThiT (blue), the S-component of the thiamine-specific ECF importer from *Lactococcus lactis* (PDB:3RLB). Hydrogen bonds are shown as dashed lines.
